# Supplementary material for: Olfactory bulb acetylcholine release dishabituates odor responses and reinstates odor investigation
Source: Nat Commun. 2018 May 14;9:1868. doi: 10.1038/s41467-018-04371-w (PMC5951802; doi:10.1038/s41467-018-04371-w)
Supplement: Supplementary file 1 — Supplementary Information [file 41467_2018_4371_MOESM1_ESM.pdf]

Supplementary Information

Supplementary Note 1: Characterization of odor investigation behavior.

We tested whether investigation times vary depending on the odorant presented. To accomplish this, we presented mice (n=5) with three different odorants (10% dilution, 1 minute duration, 3 minute ISI) in the same session. When comparing investigation times of these three odorants to that of when ethyl butyrate (E4) was presented we found no significant differences (ANOVA $F(3, 19) = 0.79$, $p = 0.51$; 2M = 31.5 ± 3.2; 2H= 33.6 ± 3.2 s; AA= 35.7 ± 4.1 s ; E4 = 29.9 ± 1.6 s; Supplementary Fig. 1c).

We next investigated the repeatability of the investigation behavior across time. As previously described, odor investigation behavior was measured for five minutes, with clean air (n=3 mice) or odor (ethyl butyrate, 10% dilution; n=4 mice) presented for the third minute only. The experiment was then repeated twenty-four hours later. A two-way repeated measures ANOVA found no effect of either trial time ($F(4,16) = 0.18$, $p = 0.95$) or day ($F(1,4) = 0.18$, $p = 0.69$) in the clean air group (Supplementary Fig. 1f). In the odor group, there was a significant effect of trial time (i.e odor presence) (2-way RM ANOVA $F(4,24)=11.81$, $p= < 0.0001$), but no effect of day ($F(1,6)=0.03$, $p=0.86$; Supplementary Fig. 1g). Further, a paired *t*-test confirmed that Minute 3 odor presentation investigation times ($t(3) = 0.21$, $p=0.84$; Day 1 = 29.55 ± 2.46 s; Day 2 = 28.18 ± 4.09 s) were not significantly different from one another across days, suggesting that this behavioral quantification is stable over time.

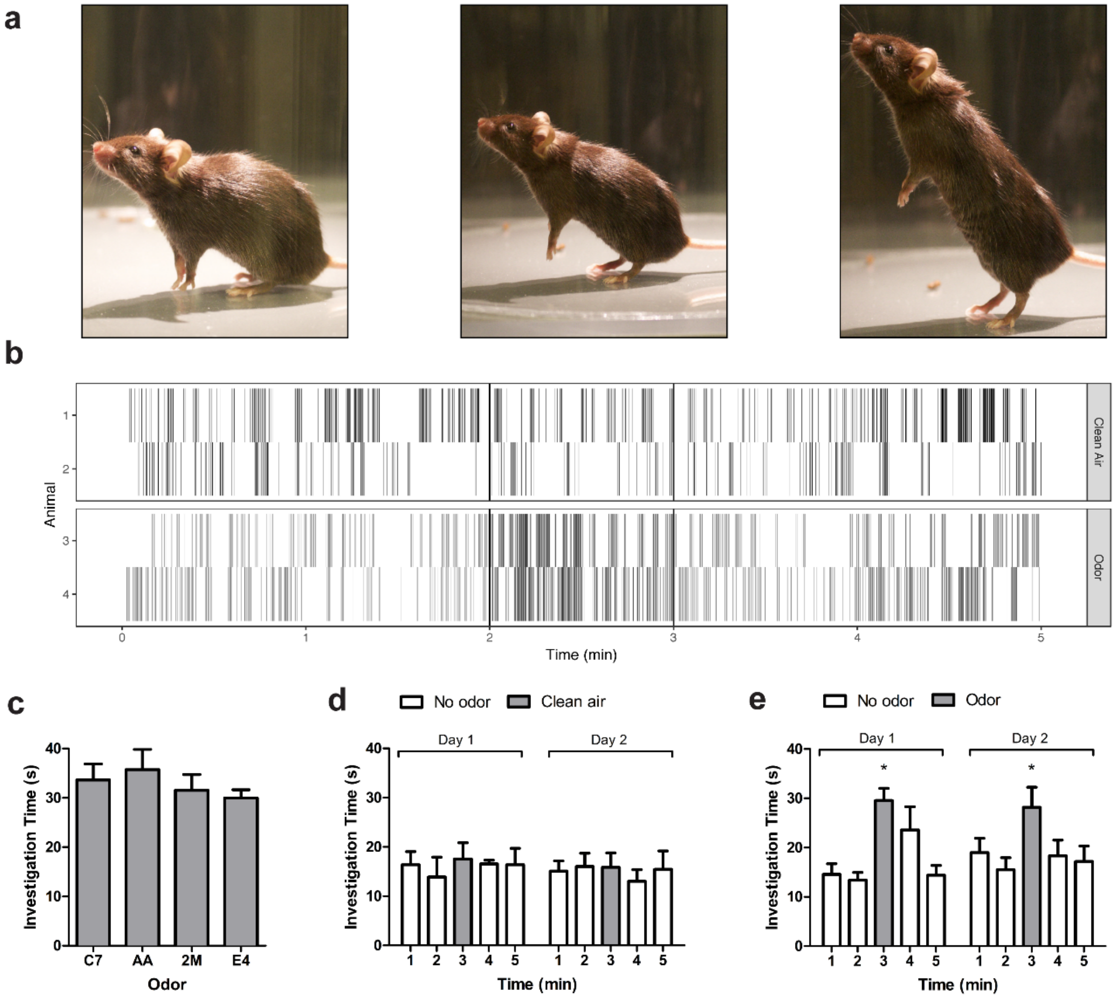

Supplementary Figure 1: Further characterization of odor investigation behavior. (a) Examples of the behaviors included in odor investigation (from right): raised-head sniffing, half-rearing, full rearing. **(b)** Example raster plots of odor investigation behavior during two minutes of no odor, a minute of clean air or odor, and another two minutes of no odor (as summarized in Fig. 3a-b). Vertical lines represent individual investigation events. **(c)** Investigation time (seconds ± SE) is similar for different odor tested, including 2-heptanone (C7), isoamyl acetate (AA), 2-methylpyrazine (2M), and ethyl butyrate (E4). **(d)** If the experiment is repeated one day later, investigation time when odor is not present is not affected, but **(e)** increases with odor onset on both days. Photos by Fletcher, ML.

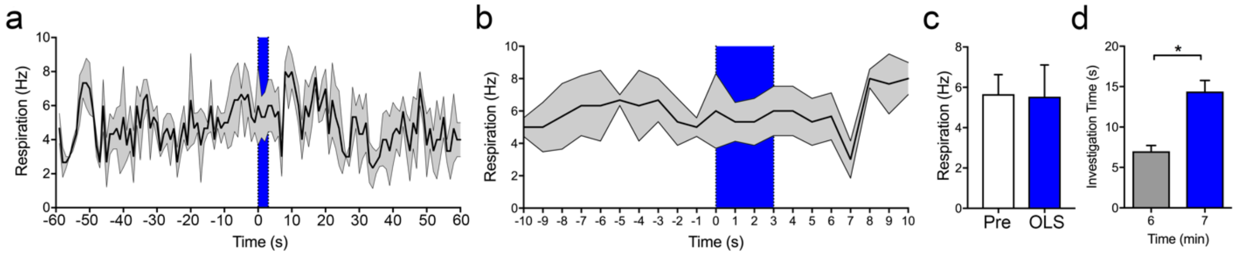

Supplementary Figure 2: OLS drives odor reinvestigation without altering sniff rates. (a) Mean respiratory rate per second centered around the OLS taken from three mice. The odor is present during the entire time period. Respiration rates are quite variable throughout the entire time. Light blue region represents time of OLS. Gray regions depict s.e.m. **(b)** Expanded view of graph from a showing no change in respiratory rate during or immediately after OLS. **(c)** Mean respiratory rates taken 3 seconds before and 3 seconds during OLS are not significantly different from one another. Paired t-test: $t(2)=0.057$, $p=0.96$. **(d)** Mean investigation times taken for the minute before (6) and after (7) OLS from the same mice. As in the original experiment, mice significantly increased their investigation time following OLS. Paired t-test: $t(2)=11.51$, $p=0.007$.
